# Effects of induced subclinical hypocalcemia in early-lactation Holstein cows without milking during infusion on parathyroid hormone and serotonin concentrations

**DOI:** 10.3168/jdsc.2024-0589

**Published:** 2024-06-13

**Authors:** W.S. Frizzarini, J.P. Campolina, M.K. Connelly, H.P. Fricke, L.L. Hernandez

**Affiliations:** 1 Department of Animal and Dairy Sciences, University of Wisconsin–Madison, Madison, WI 53706; 2 Departmento de Zootecnia, Escola de Veterinaria, Universidade Federal de Minas Gerais, Belo Horizonte, Minas Gerais, 30161-970, Brazil

## Abstract

The transition to lactation demands a substantial amount of calcium (Ca) to support colostrum and milk production. Extensive research has been focused on elucidating the interplay between the traditional Ca-parathyroid hormone-vitamin D axis and mammary-derived factors, such as serotonin (5-HT) and parathyroid-hormone-like hormone (PTHLH), in regulating Ca metabolism during the transition period. Here, we investigate the impact of induced subclinical hypocalcemia (SCH) on 5-HT and parathyroid hormone (PTH) concentrations in early-lactation dairy cows under conditions of 24-h milk stasis. Twelve multiparous Holstein cows in early lactation received either continuous intravenous infusion of saline solution or 5% ethylene glycol tetraacetic acid (EGTA) to maintain blood ionized calcium (iCa) below 1 m*M* (n = 6/treatment). Blood samples were collected hourly during infusion and 4, 8, 12, 24, and 48 h post-infusion. Urine samples were collected every 4 h during infusion and at 12, 24, and 48 h post-infusion, and milk samples were collected daily from 2 d pre-infusion to 4 d post-infusion. Infusion of EGTA resulted in decreased blood iCa during the infusion period, with iCa concentrations rebounding 24 h post-infusion. No significant treatment effects were observed on 5-HT and PTH blood concentrations. These findings underscore the importance of considering physiological distinctions in studies of Ca metabolism during the transition period.

## Highlights

• Concentrations of PTH are stabilized by 5 to 15 days in milk in response to induced subclinical hypocalcemia.
• We observed increased blood Na and hemoglobin concentrations and decreased K concentrations.
• No milking during the 24-hour EGTA challenge prevented the decrease in blood iCa observed previously at 12 hours of the challenge.

## Graphical Abstract

**Figure:**
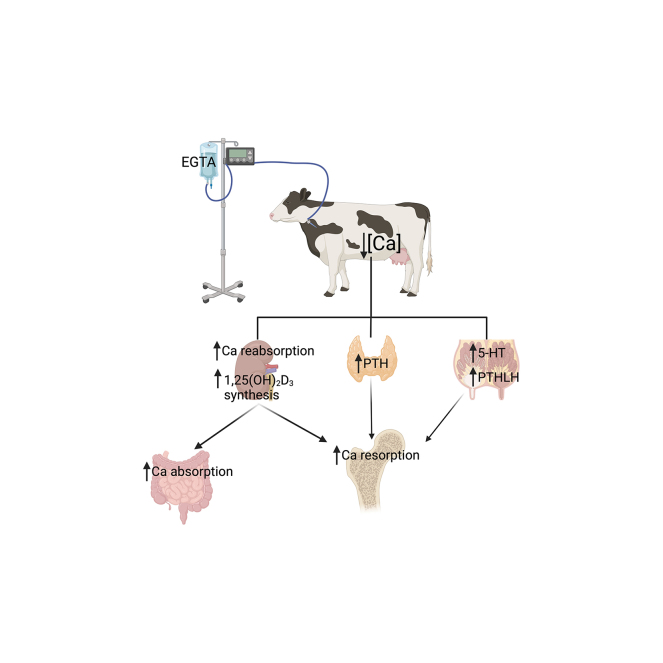
**Summary:** Multiparous cows were treated with a continuous 24-hour intravenous infusion of 0.9% sterile saline NaCl or 5% ethylene glycol tetraacetic acid (EGTA) solution dissolved in 0.9% sterile saline NaCl to maintain ionized calcium (iCa) in the subclinical hypocalcemia range, and the cows were not milked during the infusion period. We observed that PTH concentrations are not altered by EGTA challenge during 5 to 15 days in milk. Created with BioRender.com.

The onset of lactation places significant metabolic demands on dairy cows, due to the substantial calcium (Ca) influx to the mammary gland (**MG**). Consequently, maintaining adequate Ca levels is crucial for dairy cows. Subclinical hypocalcemia (**SCH**) arises from the rapid removal of Ca from the bloodstream during colostrum/milk production, surpassing the replenishment rate from bone reserves or dietary intake (Goff et al., 2002). The consequences of SCH can extend through the entire lactation and are related to displaced abomasum, metritis, mastitis, ketosis, and decreased reproductive capacity (Venjakob et al., 2021). During lactation, mammals must adapt to the high demand for Ca by increasing Ca resorption from bones, reabsorption from the kidneys, and absorption from the intestines (Greer et al., 1982). The principal hormonal regulators of Ca homeostasis are parathyroid hormone (**PTH**), 1,25-dihydroxyvitamin D [**1,25(OH)_2_D_3_**), the active form of vitamin D], and calcitonin (Greer et al., 1982).

However, the traditional Ca-PTH/1,25(OH)_2_D_3_ feedback mechanisms may be insufficient to support Ca homeostasis in early-lactation dairy cows, similar to other mammalian species. Mammary-derived endocrine signals, such as serotonin (**5-HT**) and parathyroid hormone-related protein (**PTHLH**), also play critical roles in coordinating lactation (VanHouten et al., 2003; Laporta et al., 2013). While 5-HT primarily originates from the enterochromaffin cells in the intestine, biosynthesis in the mammary epithelium during lactation has been demonstrated (Matsuda et al., 2004; Hernandez et al., 2012). The MG, acting as an accessory parathyroid gland, mobilizes Ca from bones via PTHLH during lactation. Further, research has demonstrated the disruption of the *PTHLH* gene in mammary epithelial cells decreases circulating PTHLH, reducing bone turnover (VanHouten et al., 2003; Wysolmerski, 2012). Interestingly, studies involving 5-hydroxy-l-tryptophan (**5-HTP**; a precursor of 5-HT) infusion or dietary supplementation resulted in increased circulating 5-HT and Ca concentrations, as well as enhanced mammary and PTHLH synthesis and secretion (Laporta et al., 2013; Hernández-Castellano et al., 2017; Slater et al., 2018). Infusion of 5-HTP can influence Ca metabolism, potentially redistributing Ca from circulation to the MG.

The complete mechanism by which 5-HT regulates Ca concentrations is not completely understood. It is postulated that 5-HT induces transient hypocalcemia, stimulating the negative feedback loop and resulting in increased PTHLH synthesis to restore blood Ca concentrations (Laporta et al., 2015; Connelly et al., 2021). The study herein aims to evaluate the effects of induced SCH on Ca, 5-HT, and the PTH axis in early-lactation Holstein cows that were not milked for 24 h. Further, we hypothesized that SCH and milk stasis in the MG will increase systemic 5-HT concentrations. Additionally, we hypothesized that SCH would activate the Ca feedback loop in the parathyroid gland, leading to increased PTH concentrations.

The College of Agriculture and Life Sciences Animal Care and Use Committee at the University of Wisconsin–Madison under protocol number A005316-R01 approved all procedures for this study. Animal care and use protocol guidelines were strictly followed. Cows were housed in a tiestall facility at the Dairy Cattle Center at the University of Wisconsin–Madison. Twelve multiparous (2.8 ± 0.7 lactations), nonpregnant, early-lactation (7.9 ± 2.6 DIM) Holstein cows were used in a completely randomized design (n = 6/treatment). The sample size was calculated to provide 80% power with an α of 0.05 to be able to detect a 0.2 m*M* decrease in blood Ca concentration in response to EGTA treatment. Cows were fed the common herd lactating cow TMR ad libitum, consisting of 44.7% corn silage, 30.9% alfalfa silage, 26.9% concentrate and mineral mix, and 3.62% cotton (DM = 51.8%). Cows were blocked by parity and randomly assigned to receive continuous 24 h intravenous infusion of (1) 0.9% sterile saline NaCl (n = 6, **CON**) or (2) 5% ethylene glycol tetraacetic acid (**EGTA**) solution dissolved in 0.9% sterile saline NaCl (n = 6, EGTA) to maintain ionized calcium (**iCa**) at in the SCH range (0.7 to 1.0 m*M*), and the cows were not milked during the infusion period. Each CON cow was paired with an EGTA cow to ensure equal saline volumes. The EGTA (#E3889, Sigma Aldrich, St. Louis, MO) solution was prepared according to Amundson et al. (2018) and Connelly et al. (2022).

Infusions were performed for 24 h beginning after morning milking (approximately 0630 h). Cows received 1 mL of oxytocin (20 USP units; Bimeda) before milking. A veterinary infusion pump (Heska Vet IV, Colorado) was used to maintain the infusion rate constant between the paired CON and EGTA cows. The infusion rate started at 500 mL/h, to maintain iCa between 0.7 to 1.0 m*M* (SCH). The infusion rate of the EGTA cows was modified hourly according to iCa concentration measured by the CG8+ cartridges (Abbott Laboratories), using a cow-side handheld biochemical analyzer (iStat System, Abbott Laboratories).

Jugular catheters were inserted 24 h before infusion initiation, as described previously (Connelly et al., 2022). Baseline blood samples were collected from the tail vein, 24 h before initiation of the infusion. During the infusion, blood samples were collected from the jugular catheter. Blood was then collected immediately before the start of the infusion, hourly during the infusion, 4, 8, 12, 24, and 48 h after termination of the infusion. Whole blood (**WB**) was collected into 10-mL BD Vacutainer EDTA Plus (Becton Dickinson Co., Franklin Lakes, NJ), 10-mL BD Vacutainer serum (Becton Dickinson Co.), and 10-mL lithium heparin 158 USP units (Becton Dickinson Co.) blood collection tubes and gently inverted. Immediately after the inversion, 3 to 4 mL of WB from the BD Vacutainer EDTA Plus tube was transferred to a 5-mL Eppendorf filled with approximately 35 mg of ascorbic acid (10 mg/mL) to stabilize and protect WB from 5-HT oxidation, and frozen at −20°C until further analysis (Connelly et al., 2020). Serum tubes were maintained at room temperature for 30 min. Serum and plasma tubes were centrifuged at 3,000 × *g* for 20 min at 4°C, and serum and plasma were stored at −80°C until further analysis. Urine samples were obtained immediately before infusion, every 4 h during the infusion, and 8, 12, 24, and 48 h after the end of the infusion, and stored at −20°C until further analysis. Milk samples were collected during morning and evening milking starting 2 d before infusion until 3 d after the infusion termination and stored at −20°C until further analysis.

Serotonin concentrations in milk and WB samples were analyzed according to the manufacturer's instructions (IM1749, Immunotech, Beckman Coulter) as previously described (Laporta et al., 2015; Slater et al., 2018); the intra- and interassay CV for WB 5-HT were 4.15% and 31.6%, respectively, and for milk 5-HT were 10.87% and 5.40%, respectively. Urine and milk Ca concentrations were analyzed with a colorimetric Ca assay (701220, Cayman Chemical) according to the manufacturer's instructions as previously described (Laporta et al., 2015); the intra- and interassay CV for urine Ca were 2.72% and 20.60%, respectively, and for milk Ca were 2.21% and 24.3%, respectively. Urine creatinine concentration was measured using a colorimetric assay according to manufacturer's instructions (DICT-500, QuantiChrom Creatinine Assay Kit, BioAssay System) as previously described (Slater et al., 2018); the intra- and interassay CV were 1.15% and 7.44% respectively. Before measurement of Ca concentrations in milk, these samples were mixed with 0.1 *M* acetic acid in a 1:1 proportion, and centrifuged at 13,000 × *g* for 12 min at 4°C to allow protein precipitation (Connelly et al., 2021). The supernatant was then diluted in a 1:40 proportion and analyzed for Ca concentration.

Data were analyzed with the MIXED procedure of SAS (version 9.4, SAS Institute Inc., Cary, NC). A baseline measurement was determined using the sample collected 24 h before the infusion started and it was used as a covariate. Fixed terms in the model for blood gases and minerals, PTH, urine Ca, WB 5-HT, milk 5-HT, and milk Ca were treatment, lactation, time, block, and interaction of treatment × time. For milk yield and DMI, the same variables were used as fixed terms with the addition of DIM. Fixed terms in the model for infusion rate were time and block. The random statement in all models included cow and time was considered the repeated measure. The covariance structure used for blood gases and minerals, PTH, urine Ca, and WB 5-HT was spatial power due to different sampling timeframes. For the daily variables such as milk 5-HT and Ca, milk yield, and DMI, the AR(1) structure was used to account for autocorrelated errors. The residuals from the models were analyzed for normality, and when the normality assumption failed, data were transformed according to diagnostic plots. Least squares means are reported for each variable, and statistical significance was declared if *P* ≤ 0.05. Mean comparisons for all measurements were performed using the Tukey test when treatment or treatment × time effects were significant.

Treatment (*P* = 0.01) and treatment × time (*P* < 0.01) effects were observed for iCa concentrations (Figure 1A; Table 1). Overall mean iCa concentration was 1.3 ± 0.03 and 1.0 ± 0.03 mmol/L for CON and EGTA, respectively. The EGTA-treated cows had lower iCa concentrations than CON cows from 1 h until 24 h during infusion. However, 4 h after infusion end iCa concentrations equalized, and 24 h post-infusion, EGTA cows had higher iCa concentrations than CON cows (1.4 ± 0.05 and 1.2 ± 0.05 mmol/L, respectively). Treatment (*P* = 0.02) and treatment × time (*P* < 0.01) effects were observed for urine Ca concentrations (Figure 1B). The overall mean was 0.02 ± 0.002 and 0.01 ± 0.002 mg Ca/mg creatinine for CON and EGTA, respectively. The EGTA-infused cows exhibited the lowest urine Ca concentrations than CON cows during the infusion, but 8 h post-infusion, concentrations were similar. No treatment (*P* = 0.3) or treatment × time (*P* = 0.5) effects were detected for milk Ca concentrations (Figure 1C). The EGTA-treated cows had higher hemoglobin concentrations (*P* = 0.02; 8.9 ± 0.1 and 8.4 ± 0.1 g/dL, for EGTA and CON respectively; Table 1), hematocrit percentage (*P* = 0.02; 26.1 ± 0.2 and 24.8 ± 0.2% packed cell volume for EGTA and CON, respectively; Table 1), and sodium (Na) concentrations (*P* = 0.01; 141.4 ± 0.2 and 139.9 ± 0.2, for EGTA and CON respectively; Table 1) than CON cows. The EGTA-treated cows had lower potassium (K) concentrations than CON cows (*P* = 0.01; 3.7 ± 0.03 and 3.9 ± 0.03 mmol/L; Table 1). No treatment effects were observed in WB 5-HT (*P* = 0.7, 2,519.7 ± 186.2 and 2,408 ± 185.8 ng/mL for CON and EGTA respectively; Figure 2A) or milk 5-HT concentrations (*P* = 0.7; 2.7 ± 0.7 and 2.9 ± 0.7 ng/mL for CON and EGTA respectively; Figure 2B). No treatment effect was detected for PTH concentrations (*P* = 0.7; 243.3 ± 16.1 and 234.7 ± 16.4 pg/mL for CON and EGTA, respectively; Figure 1D). Time effect (*P* < 0.01) was observed for plasma PTH concentrations, with the highest concentration at 36 h after the beginning of infusion and the lowest at 0 h (341.4 ± 25.2 and 174.1 ± 25.2 pg/mL, respectively; Figure 1D). The infusion rate was highest at 23 h after the beginning of infusion, and lowest at 8 h after the beginning of infusion (*P* < 0.01; 758.0 ± 63.8 and 162.5 ± 63.8 mL/h, respectively; Figure 1E).

**Figure 1:**
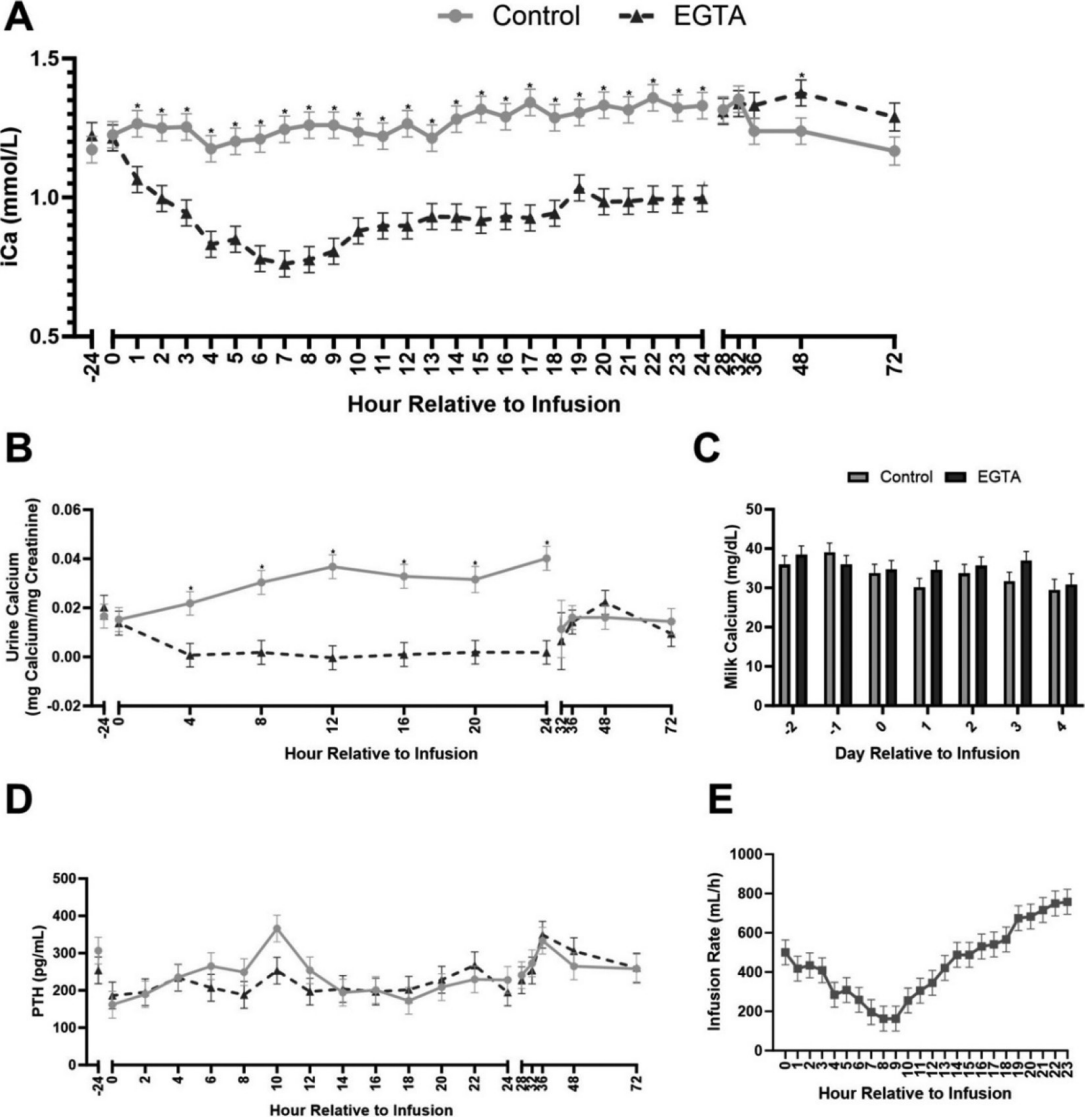
Whole-blood ionized calcium (iCa) concentrations (A), urine calcium concentrations (B), milk calcium concentrations (C), plasma parathyroid hormone (PTH; D), and hourly infusion rates across the 24-h infusion period (E) of multiparous early-lactation cows infused with 5% ethylene glycol tetraacetic acid (EGTA) or saline (control) for 24 h. Ionized calcium concentrations: treatment (*P* < 0.01), time (*P* < 0.01), and treatment × time (*P* < 0.01) effects. Urine calcium concentrations: treatment (*P* < 0.01), block (*P* = 0.01), and treatment × time (*P* < 0.01) effects. Plasma PTH concentrations: time (*P* < 0.01) effect. Infusion rate: time (*P* < 0.01) and block (*P* < 0.01) effects. Data are presented as individual values ± SEM.

**Table 1:** Effect of induced subclinical hypocalcemia on acid-base status and blood parameters in multiparous Holsteins cows (LSM ± SE)

Item^1^	Treatment (Trt)		*P*-value							
	CON	EGTA	Trt	Time	Day	Lactation	Block	Trt × Time	Day × Trt	DIM
tCO_2_ (m*M*)	28.7 ± 0.3	26.6 ± 0.3	<0.01	0.1	—	0.3	<0.01	0.1	—	—
pCO_2_ (mmHg)	41.6 ± 0.4	40.6 ± 0.6	0.2	0.2	—	0.4	<0.01	0.4	—	—
sO_2_ (%)	81.4 ± 1.8	78.4 ± 2.2	0.4	0.02	—	0.5	<0.01	0.3	—	—
pO_2_ (mmHg)	40.5 ± 10.6	66.0 ± 7.3	0.4	0.03	—	0.8	<0.01	0.4	—	—
Hct (% PCV)	24.9 ± 0.2	26.5 ± 0.1	<0.01	<0.01	—	<0.01	<0.01	0.01	—	—
HCO_3_ (m*M*)	27.4 ± 0.3	25.4 ± 0.3	<0.01	0.1	—	0.3	<0.01	0.1	—	—
Hgb (g/dL)	8.5 ± 0.1	9.0 ± 0.1	<0.01	<0.01	—	<0.01	<0.01	0.01	—	—
pH	7.4 ± 0.003	7.4 ± 0.003	<0.01	0.03	—	0.05	<0.01	0.2	—	—
BE (m*M*)	3.0 ± 0.3	0.8 ± 0.3	<0.01	0.05	—	0.2	<0.01	0.1	—	—
Na (m*M*)	139.9 ± 0.2	141.8 ± 0.2	<0.01	<0.01	—	0.2	0.3	<0.01	—	—
K (m*M*)	3.9 ± 0.03	3.6 ± 0.03	<0.01	<0.01	—	0.03	<0.01	0.1	—	—
iCa (m*M*)	1.6 ± 0.03	0.9 ± 0.03	<0.01	<0.01	—	0.7	0.2	<0.01	—	—
Glucose (mg/dL)	61.6 ± 0.8	62.33 ± 0.85	0.6	<0.01	—	0.1	<0.01	0.8	—	—
Milk yield pre-infusion (kg)	35.0 ± 1.8	35.7 ± 2.4	0.8	—	0.01	0.1	0.5	—	0.8	0.02
Milk yield post-infusion (kg)	44.0 ± 4.2	46.4 ± 5.6	0.8	—	0.6	0.6	0.7	—	0.5	0.6
DMI (kg)	19.1 ± 0.9	19.3 ± 0.9	0.9	—	0.03	0.1	0.6	—	0.7	0.6

1 tCO_2_ = total CO_2_; pCO_2_ = CO_2_ partial pressure; sO_2_ = O_2_ saturation; pO_2_ = O_2_ partial pressure; Hct = hematocrit; PCV = packed cell volume; HCO_3_ = bicarbonate; Hgb = hemoglobin; BE = base excess; iCa = ionized calcium.

**Figure 2:**
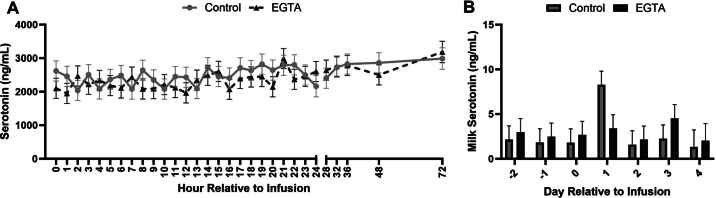
Concentrations of whole-blood (WB) serotonin across the experimental period (A) and milk serotonin concentrations (B) of multiparous early-lactation cows infused with 5% ethylene glycol tetraacetic acid (EGTA) or saline (control) for 24 h. Whole blood serotonin concentrations: time (*P* < 0.01) effect. Milk serotonin concentrations: day (*P* = 0.01) and block (*P* = 0.02) effects. Data are presented as individual values ± SEM.

Previous research documented the triphasic pattern of hypocalcemia induced by Na_2_EDTA infusions in lactating and dry cows and calves, characterized by a rapid decline in Ca concentrations followed by a plateau, succeeded by a secondary drop (Ramberg et al., 1967; Desmecht et al., 1995). Although employing a distinct methodology, our study showed this multiphasic response to EGTA infusion, consistent with the findings of Connelly et al. (2022). In both studies, the initial decline in Ca concentrations occurred at 4 h of infusion initiation, which corresponds to “phase 1” observed in the study by Ramberg et al. (1967). Notably, Connelly et al. (2022) reported a slight reduction in iCa around 12 h of infusion, coinciding with milking, an observation not replicated in our study, as cows were not milked during the infusion period, indicating Ca loss due to milk removal and subsequent refilling of the gland. Goff et al. (2002) underscored the importance of milk production on Ca concentration by demonstrating the prevention of hypocalcemia after parturition using mastectomy in cows. Furthermore, Connelly et al. (2022) noted that dry cows infused with EGTA exhibited more stable iCa concentrations and required less EGTA to induce SCH compared with early-lactation cows, exhibiting the importance of considering such differential responses in studies of Ca metabolism. In our study, cows receiving EGTA had decreased urine Ca concentrations during the infusion period, aligning with findings from previous investigations using EGTA or Na_2_EDTA to induce hypocalcemia (Schonewille et al., 1999; Connelly et al., 2022).

The decrease in blood K concentration may be due to issues linked to hypocalcemia, such as recumbency, stress, and excitement associated with muscle weakness and recumbency (Daniel, 1980). Recumbency-induced stress could increase the concentration of ACTH, therefore increasing glucocorticoids with mineralocorticoid activity, causing a decrease in K concentration (Daniel, 1980). Metabolic acidosis, indicated by reductions in blood HCO_3_, pH, and tCO_2_ during the EGTA infusion period, aligns with earlier findings (Desmecht et al., 1995; Connelly et al., 2022). Similarly, a study in calves receiving Na_2_EDTA infusion reported increased hemoglobin concentrations, attributed to the formation of circulating Ca-EDTA complexes, potentially explaining our observations (Desmecht et al., 1995).

Serotonin has been proven to be a pivotal regulator of lactation homeostasis, with direct associations with Ca regulation during lactation. Studies in rodent and dairy cows have highlighted 5-HT's production by the MG during lactation and its significant concentration increase compared with nonlactating counterparts (Pai and Horseman, 2008; Weaver et al., 2016; Connelly et al., 2022). In mice it was shown that 5-HT induces PTHLH secretion in the MG, suggesting the existence of a 5-HT-PTHLH axis that contributes to Ca mobilization by the MG (Hernandez et al., 2012). In a recent study using early-lactation (5 to 15 DIM) and dry cows infused with EGTA or saline for 24 h to induce SCH, the authors did not find differences in 5-HT concentrations between cows receiving EGTA or saline infusion; however, they detected an increase in 5-HT concentrations in early-lactation cows compared with the dry cows (Connelly et al., 2022). Similarly, in the present study no difference was detected between EGTA and CON, and 5-HT concentrations in the blood were similar to Connelly et al. (2022). Previous studies have shown that when 5-HT is manipulated by 5-HTP administration during lactation, circulating 5-HT increases, whereas Ca concentration in the blood decreases, showing a direct effect of 5-HT on Ca regulation (Laporta et al., 2015; Connelly et al., 2021). However, this experiment and others have shown that when Ca is manipulated by EGTA infusion in lactating and dry cows it does not affect 5-HT concentrations (Amundson et al., 2018; Connelly et al., 2022).

Cows infused with Na_2_EDTA had decreased Ca concentrations and increased PTH concentrations up to 3 to 4 times higher than the control during maximal stimulation, and this increase in PTH concentrations persisted beyond the infusion period (Sherwood et al., 1966; Ramberg et al., 1967). However, the second infusion with Na_2_EDTA after Ca infusion did not produce the same results as the PTH response was less dramatic than the first EDTA infusion (Sherwood et al., 1966). They suggested that Ca infusion delayed the parathyroid gland's restoration of PTH synthesis following the suppression of the gland (Sherwood et al., 1966). In a prospective study using 89 multiparous cows, they measured Ca concentration on 1 and 4 DIM and found an effect of time for PTH concentration with the peak occurring at 1 DIM (Seely and McArt, 2023). Similarly, Seely and McArt (2023) found an effect of time on PTH concentrations, with the peak observed on 1 DIM. Chandler et al. (2023) challenged early-lactation cows with LPS and one group of cows received intravenous Ca infusion for 12 h and the other group received saline infusion. The authors observed an increase in tCa concentrations for the group receiving Ca, but they did not find differences in PTH concentrations during the infusion. Taken together, these studies along with the suppression of PTH synthesis suggested by Sherwood et al. (1966), may explain why PTH concentrations did not increase in cows subjected to the EGTA infusion in the present study. The cows in the present study were between 5 to 15 DIM, and their Ca nadir had already occurred at parturition. This suggests that cows had already activated their Ca feedback mechanisms, which corresponded to the Ca infusion in Sherwood et al. (1966). Rather, it is possible that induction of SCH with EGTA in early lactation corresponded to the second Na_2_EDTA infusion seen in Sherwood et al. (1966). These findings suggest while PTH did not respond as expected to the EGTA challenge, it is possible that additional Ca regulation mechanisms are being activated. Further, it is possible that PTHLH was partly responsible for the maintenance of Ca homeostasis during early lactation, but we cannot confirm this because PTHLH was not able to be measured.

Our study contributes to the growing literature elucidating the complex interplay between Ca regulation, 5-HT signaling, and lactation dynamics. Our findings show the importance of considering physiological nuances in studies of Ca homeostasis during the periparturient period. Further investigations are warranted to advance our understanding of Ca regulation in dairy cows.

## References

[bib1] Amundson L.A., Rowson A.D., Crump P.M., Prichard A.P., Cheng A.A., Wimmler C.E., Klister M., Weaver S.R., Bascom S.S., Nuzback D.E., Zanzalari K.P., Hernandez L.L. (2018). Effect of induced hypocalcemia in nonlactating, nonpregnant Holstein cows fed negative DCAD with low, medium, or high concentrations of calcium. J. Anim. Sci..

[bib2] Chandler T.L., Westhoff T.A., LaPierre P.A., Frizzarini W., Hernandez L.L., Overton T.R., Mann S. (2023). Eucalcemia during lipopolysaccharide challenge in postpartum dairy cows: II. Calcium dynamics. J. Dairy Sci..

[bib3] Connelly M.K., Henschel S.R., Kuehnl J.M., Cheng A.A., Nashold F., Hernandez L.L. (2022). Physiological adaptations in early-lactation cows result in differential responses to calcium perturbation relative to nonlactating, nonpregnant cows. J. Dairy Sci..

[bib4] Connelly M.K., Marshall A.M., Crump P.M., Hernandez L.L. (2020). Short communication: The effect of ruminal administration of 5-hydroxy-L-tryptophan on circulating serotonin concentrations. J. Dairy Sci..

[bib5] Connelly M.K., Weaver S.R., Kuehnl J.M., Fricke H.P., Klister M., Hernandez L. (2021). Elevated serotonin coordinates mammary metabolism in dairy cows. Physiol. Rep..

[bib6] Daniel R.C.W. (1980). Induced hypocalcaemia in cows and sheep II. Changes in plasma potassium levels. Br. Vet. J..

[bib7] Desmecht D.J.-M., Linden A.S., Godeau J.-M., Lekeux P.M. (1995). Experimental production of hypocalcemia by EDTA infusion in calves: A critical appraisal assessed from the profile of blood chemicals and enzymes. Comp. Biochem. Physiol. A Comp. Physiol..

[bib8] Goff J.P., Kimura K., Horst R.L. (2002). Effect of mastectomy on milk fever, energy, and vitamins A, E, and β-carotene status at parturition. J. Dairy Sci..

[bib9] Greer F.R., Tsang R., Searcy J., Levin R., Steichen J. (1982). Mineral homeostasis during lactation—Relationship to serum 1,25-dihydroxyvitamin D, 25-hydroxyvitamin D, parathyroid hormone, and calcitonin. Am. J. Clin. Nutr..

[bib10] Hernandez L.L., Gregerson K.A., Horseman N.D. (2012). Mammary gland serotonin regulates parathyroid hormone-related protein and other bone-related signals. Am. J. Physiol. Endocrinol. Metab..

[bib11] Hernández-Castellano L.E., Hernandez L.L., Sauerwein H., Bruckmaier R.M. (2017). Endocrine and metabolic changes in transition dairy cows are affected by prepartum infusions of a serotonin precursor. J. Dairy Sci..

[bib12] Laporta J., Moore S.A.E., Weaver S.R., Cronick C.M., Olsen M., Prichard A.P., Schnell B.P., Crenshaw T.D., Peñagaricano F., Bruckmaier R.M., Hernandez L.L. (2015). Increasing serotonin concentrations alter calcium and energy metabolism in dairy cows. J. Endocrinol..

[bib13] Laporta J., Peters T.L., Weaver S.R., Merriman K.E., Hernandez L.L. (2013). Feeding 5-hydroxy-L-tryptophan during the transition from pregnancy to lactation increases calcium mobilization from bone in rats. Domest. Anim. Endocrinol..

[bib14] Matsuda M., Imaoka T., Vomachka A.J., Gudelsky G.A., Hou Z., Mistry M., Bailey J.P., Nieport K.M., Walther D.J., Bader M., Horseman N.D. (2004). Serotonin regulates mammary gland development via an autocrine-paracrine loop. Dev. Cell.

[bib15] Pai V.P., Horseman N.D. (2008). Biphasic regulation of mammary epithelial resistance by serotonin through activation of multiple pathways. J. Biol. Chem..

[bib16] Ramberg C.F., Mayer G.P., Kronfeld D.S., Aurbach G.D., Sherwood L.M., Potts J.T. (1967). Plasma calcium and parathyroid hormone responses to EDTA infusion in the cow. Am. J. Physiol..

[bib17] Schonewille J.T., Van't Klooster A.T., Wouterse H., Beynen A.C. (1999). Hypocalcemia induced by intravenous administration of disodium ethylenediaminotetraacetate and its effects on excretion of calcium in urine of cows fed a high chloride diet. J. Dairy Sci..

[bib18] Seely C.R., McArt J.A.A. (2023). Circulating parathyroid hormone and serotonin in multiparous cows with differing postparturient serum calcium concentrations. J. Dairy Sci..

[bib19] Sherwood L.M., Potts J.T., Care A.D., Mayer G.P., Aurbach G.D. (1966). Evaluation by radioimmunoassay of factors controlling the secretion of parathyroid hormone. Nature.

[bib20] Slater C.J., Endres E.L., Weaver S.R., Cheng A.A., Lauber M.R., Endres S.F., Olstad E., DeBruin A., Crump P.M., Block E., Hernandez L.L. (2018). Interaction of 5-hydroxy-l-tryptophan and negative dietary cation-anion difference on calcium homeostasis in multiparous peripartum dairy cows. J. Dairy Sci..

[bib21] VanHouten J.N., Dann P., Stewart A.F., Watson C.J., Pollak M., Karaplis A.C., Wysolmerski J.J. (2003). Mammary-specific deletion of parathyroid hormone–related protein preserves bone mass during lactation. J. Clin. Invest..

[bib22] Venjakob P.L., Staufenbiel R., Heuwieser W., Borchardt S. (2021). Association between serum calcium dynamics around parturition and common postpartum diseases in dairy cows. J. Dairy Sci..

[bib23] Weaver S.R., Prichard A.P., Endres E.L., Newhouse S.A., Peters T.L., Crump P.M., Akins M.S., Crenshaw T.D., Bruckmaier R.M., Hernandez L.L. (2016). Elevation of circulating serotonin improves calcium dynamics in the peripartum dairy cow. J. Endocrinol..

[bib24] Wysolmerski J.J. (2012). Parathyroid hormone-related protein: An update. J. Clin. Endocrinol. Metab..

